# Midkine is a serum and urinary biomarker for the detection and prognosis of non-small cell lung cancer

**DOI:** 10.18632/oncotarget.13865

**Published:** 2016-12-10

**Authors:** Xin Xia, Jian-Jun Lu, Shui-Shen Zhang, Chun-Hua Su, Hong-He Luo

**Affiliations:** ^1^ Department of Thoracic Surgery, The First Affiliated Hospital, Sun Yat-Sen University, Guangzhou, People's Republic of China

**Keywords:** midkine, non-small cell lung cancer (NSCLC), serum biomarker, urine biomarker, survival

## Abstract

Midkine, a heparin-binding growth factor, has been identified as a promising cancer biomarker. In non-small cell lung cancer (NSCLC), the serum and urine midkine levels have not been intensively investigated. The aim of the present study was to investigate the diagnostic and prognostic potential of serum and urine midkine levels in patients with NSCLC. The serum midkine levels were measured in 153 patients with NSCLC, 23 patients with benign pulmonary disease and 95 healthy controls using ELISA. Urine midkine levels were examined in 20 controls and 45 patients with NSCLC. Midkine expression in tumor tissues from 72 patients with NSCLC who underwent definitive surgical resection without any pre-operative treatments was examined by immunohistochemistry. Serum levels were significantly higher in patients with NSCLC than in healthy controls (657.36±496.58 pg/ml vs. 194.49±122.57 pg/ml, *P*<0.001). As shown in the ROC curve analysis, the sensitivity and specificity of the cut-off serum midkine concentration of 400 pg/ml for predicting the presence of NSCLC were 71.2% and 88.1%, respectively. Positive correlations between the serum midkine levels and immunohistochemistry staining scores (r=0.315, *P*=0.007) and between the serum midkine levels and urine midkine levels (r=0.636, *P*<0.001) were observed using Spearman's bivariate correlations. The serum midkine concentration was identified as an independent prognostic factor by multivariate analysis, and its overexpression yielded a relative risk of death of 2.072 (0.01<*P*<0.05, 95%CI: 1.104-3.890). Thus, the serum and urine midkine levels may be useful, minimally invasive biomarkers for detecting and predicting the prognosis of NSCLC.

## INTRODUCTION

Lung cancer is the leading type of cancer and cause of cancer mortality worldwide. The incidence of non-small cell lung cancer (NSCLC), a major form of lung cancer, has increased in the past several decades [1]. Despite advancements in diagnosis and therapeutic strategies of lung cancer, only 15% survive more than 5 years in patients with NSCLC [2]. Concerns about the detection of early stage lung cancer have become an inevitable issue in decisions regarding treatment. Additionally, many recent studies have focused on the development of detection techniques that are specific, convenient and non-invasive.

Midkine, a heparin-binding growth factor, was initially identified as the product of a retinoic acid-responsive gene expressed during embryogenesis [3]. The biological activities of midkine in malignant tumors include proliferation, angiogenesis, invasion and metastasis [4]. Various cancers express significantly higher levels of the midkine protein in early stage tumor tissues than in adjacent normal tissues [5–8]. Because midkine is a secreted protein and enzyme immunoassays have been developed to detect this protein, an increase in the serum and urinary midkine levels has been reported in patients with various tumors [9, 10]. Thus, serum and urinary midkine levels are potentially important tools for the surveillance and early diagnosis of malignancies.

Numerous investigations have revealed an association between midkine expression in malignant tumors with the clinicopathological and prognostic significance [11, 12]. Correlations between the expression of midkine protein and the serum midkine concentrations have been established in esophageal cancer and head and neck squamous cancer [13, 14]. Recently, Yuan et al. reported a significant association between the overexpression of midkine mRNA and protein and the malignant status and poor prognosis of patients with NSCLC [15]. However, the diagnostic and prognostic value of the serum and urine midkine levels for patients with NSCLC, especially the urine midkine levels, have not yet been extensively studied.

In this study, we measured the midkine concentrations in the serum and urine from patients with NSCLC or benign pulmonary diseases and from healthy individuals. We examined NSCLC tissues using immunohistochemistry to determine the origin of the midkine protein. The potential prognostic value of the serum midkine levels was evaluated in a comparison with conventional tumor markers, such as neuron-specific enolase (NSE), carcinoembryonic antigen (CEA) and cytokeratin 19 fragment (CYFRA21-1). Therefore, our current study intended to systematically evaluate the serum and urine midkine levels in patients with NSCLC to determine their diagnostic and prognostic value.

## RESULTS

### Correlation between midkine levels and clinicopathological characteristics in patients with NSCLC

**Figure 1:**
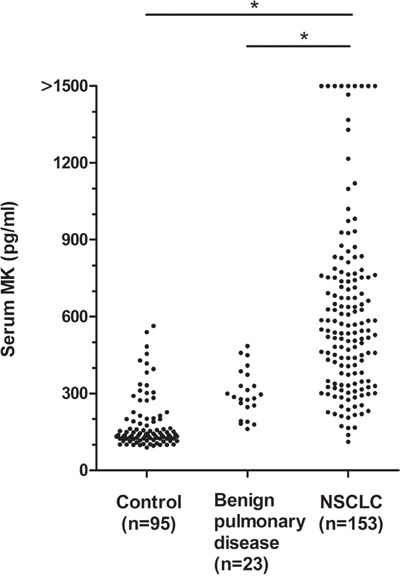
Serum midkine concentrations in patients with NSCLC, benign pulmonary diseases and control individuals The statistical significance of the differences was determined using the Mann-Whitney *U* test,*P<0.001.

The serum midkine levels in 153 patients with NSCLC, 23 patients with benign pulmonary diseases and 95 healthy controls were analyzed using an ELISA to investigate the role of midkine as a tumor marker for NSCLC. The median serum midkine level in patients with NSCLC (544.0 pg/ml; range, 110.7-2957.7 pg/ml) was significantly increased compared with healthy individuals (147.6 pg/ml; range, 85.8-581.5 pg/ml, 0.01<*P*<0.05) and patients with benign pulmonary diseases (286.0 pg/ml; range, 161.4-485.3 pg/ml, 0.01<*P*<0.05) (Figure 1). There were no correlations between the serum midkine concentrations and clinicopathological parameters (Table 1).

**Table 1:** Clinical variables of 153 patients with NSCLC

Variables		No. of patients	Serum midkine(pg/ml)	*P* value
**Gender**	MaleFemale	9954	701±574576±297	0.550^1^
**Age**	≤50>50	42111	603±489678±499	0.318^1^
**Smoking**	YesNo	8964	721±537642±463	0.653^1^
**Pathology classification**	AdenocarcinomaSquamous cell carcinoma	9162	673±530633±446	0.921^1^
**Differentiated degree**	High and middleLow and undifferentiated	7974	577±304742±632	0.482^1^
**T classification^2^**	T0-2T3-4	10449	653±557666±339	0.087^1^
**N classification^2^**	N0-1N2-3	10548	657±489657±517	0.307^1^
**M classification^2^**	M0M1	13914	681±508421±285	0.011^1^
**Clinical stage^2^**	I-IIIII-IV	8766	657±525658±461	0.464^1^

^1^Mann–Whitney *U* test.

^2^clinical staging of TNM classification.

**Figure 2:**
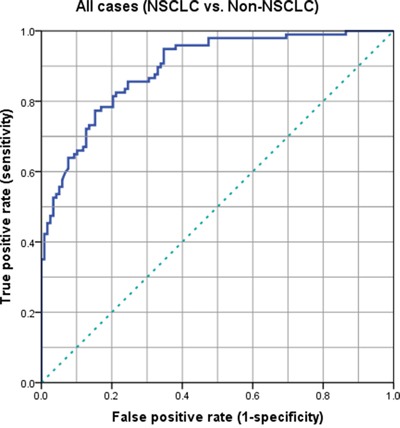
ROC curves for patients with NSCLC and controls who did not have NSCLC

Based on our data, the appropriate cut-off value of serum midkine levels for predicting the presence of NSCLC was 400 pg/ml (Figure 2). As shown in the receiver operating characteristic (ROC) curve analysis, at a cut-off value of 400 pg/ml, the sensitivity, specificity, positive predictive value, negative predictive value, and accuracy of the serum midkine levels for identifying patients with NSCLC were 71.2%, 88.1%, 90.3%, 82.5% and 86.7%, respectively. We also investigated the sensitivity and specificity of three conventional tumor markers, CEA, NSE and CYFRA21-1. The sensitivity and specificity of the serum midkine levels were significantly higher than CEA (42.0% and 70.6%, respectively), NSE (44.4% and 68.1%, respectively) and CYFRA21-1 (39.4% and 72.3%, respectively) (Table 2).

**Table 2:** Sensitivity and specificity of serum midkine and conventional tumor markers

	CEA	NSE	CYFRA21-1	Midkine	*P*^1^
**Sensitivity**	42.0%	44.4%	39.4%	71.2%	<0.05
**Specificity**	70.6%	68.1%	72.3%	88.1%	<0.05

^1^ Fisher's exact test.

### Correlation between the serum midkine concentrations and immunohistochemistry analysis of midkine expression in tumor tissues

**Figure 3:**
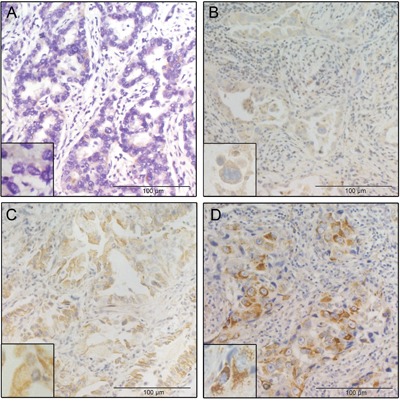
Representative IHC results of tumor specimens with different intensities of midkine staining **A**. The mean IHC score was 0. **B**. The mean IHC score was 1. **C**. The mean IHC score was 8. **D**. The mean IHC score was 9.

**Figure 4:**
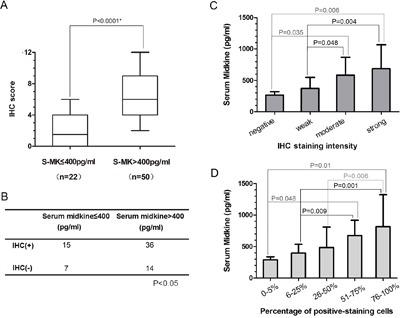
Midkine immunoreactivity and serum midkine levels in 72 patients with NSCLC The mean IHC scores and standard deviations are shown for the high-serum and low-serum midkine expression groups. The statistical significance of the differences was determined using the Mann-Whitney *U* test. **A**. Association between the serum midkine levels and immunoreactivity. The statistical significance of the differences was determined using the Spearman's correlation test (r=0.315, *P*=0.007). **B**. Serum midkine levels were significantly elevated in patients with IHC staining scores of 0 to 3 (F=4.720, *P*=0.005). **C**. and IHC proportion scores of 0 to 4 (F=4.512, *P*=0.003). **D**.

We examined the expression of the midkine protein in 72 samples collected during the surgical resection of NSCLC in patients without preoperative treatment using immunohistochemistry (IHC). Representative IHC results are presented to show tumor specimens with different levels of midkine expression (Figure 3). The mean IHC staining score in patients with high serum midkine values (range: 0-6, median: 1.5) was significantly higher than the score in patients with low serum midkine values (range: 2-12, median: 6.0, *P*<0.0001, Figure 4A). Spearman's bivariate correlations showed positive correlations between the serum midkine levels and IHC staining scores (r=0.315, *P*=0.007, Figure 4B). The serum midkine level was significantly increased in tissues with an IHC staining score of 0 to 3 (F=4.720, *P*=0.005, Figure 4C) and an IHC proportion score of 0 to 4 (F=4.512, *P*=0.003, Figure 4D).

### Urinary midkine levels are useful in the diagnosis of NSCLC cases

**Figure 5:**
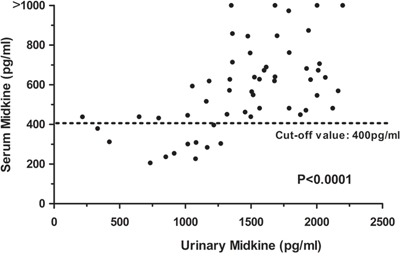
Relationship between the serum midkine concentrations and urine midkine concentrations in 72 NCSCLC patients The statistical significance of the differences was determined using the Spearman's correlation test (r=0.636, *P*<0.0001).

We measured the midkine levels in the urine from 45 patients and 20 healthy controls to determine whether the urinary midkine levels may be used as a non-invasive biomarker for detecting NSCLC. Consistent with the serum analysis, the urine midkine levels were significantly increased in patients with NSCLC compared with cancer-free individuals (0.01<*P*<0.05, data not shown). Additionally, the urine midkine levels were significantly associated with the serum midkine levels in patients with NSCLC (Spearman's correlation coefficient: r=0.636, *P*<0.001, Figure 5).

### Serum midkine levels predict survival in NSCLC cases

**Figure 6:**
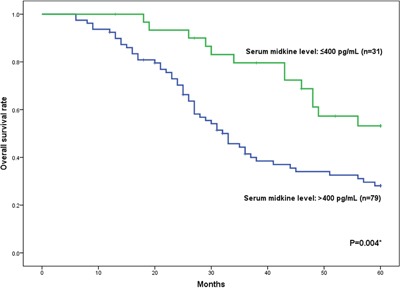
Overall survival rate of 110 patients with NSCLC who underwent surgery The low-serum midkine group includes patients with serum midkine levels ≤400 pg/ml (n=31). The high-serum midkine group includes patients with serum midkine levels >400 pg/ml (n=79). The statistical significance of the differences was determined using the log-rank test (*P*=0.004).

The correlations between survival and the serum midkine levels in patients with NSCLC were investigated using the Kaplan–Meier method. One hundred ten patients who received radical lobectomy and systematic lymphadenectomy were followed for 5 years. In present study, a univariate analysis was applied to determine the prognostic value of the serum midkine concentrations and other clinical characteristics (Table 3). When the patients’ serum midkine concentrations, clinical stages, genders, and ages were assessed with a multivariate analysis using Cox's proportional hazards model, the serum midkine concentration was identified as an independent prognostic factor (Table 4). The survival rate in the high-serum midkine group (n=79) was significantly worse than the low-serum midkine group (n=31, 28.1% vs. 53.2%). Moreover, the 95% confidence intervals (CIs) were 17.7%-38.5% and 34.4%-72.0%, respectively (0.01<*P*<0.05, Figure 6). The relative risk of death for serum midkine values more than 400 pg/ml was 2.072 (95% CI: 1.104 - 3.890).

**Table 3:** Univariate analysis of patients with NSCLC undergoing lobectomy (n = 110)

	*P* value	Hazard ratio	95%CI^1^
T classification^2^ T0-2 vs. T3-4	0.112	1.672	0.887-3.154
N classification^2^ N0-1 vs. N2-3	0.792	1.158	0.389-3.448
Clinical stage^2^ 0-II vs. IIIa	0.335	1.628	0.604-3.448
Serum midkine ≤400 vs. >400 (pg/ml)	0.023	2.072	1.104-3.890

^1^95% CI, 95% confidence interval

^2^pathologic staging of TNM classification

**Table 4:** Multivariate analysis of the survival of patients with NSCLC undergoing lobectomy (n = 110)

	*P* value	Hazard ratio	95%CI^1^
T classification^2^ T0-2 vs. T3-4	0.112	1.672	0.887-3.154
N classification^2^ N0-1 vs. N2-3	0.792	1.158	0.389-3.448
Clinical stage^2^ 0-II vs. IIIa	0.335	1.628	0.604-3.448
Serum midkine .400 vs. >400 (pg/ml)	0.023	2.072	1.104-3.890

## DISCUSSION

In this study, the serum and urinary midkine concentrations were significantly elevated in patients with NSCLC compared with healthy normal samples and patients with non-malignant pulmonary diseases, consistent with a report on the use of midkine levels for determining the prognosis of patients with NSCLC by Yuan et al. [15]. The sensitivity of the ability of the serum midkine levels to detect NSCLC was 85.6% at a cut-off value of 400 pg/ ml, with a specificity of 88.1%. Additionally, there was a significant correlation between the serum and urinary midkine levels. This study is the first to show that measurements of the serum and urine midkine levels may be a beneficial and minimally invasive method to diagnose and determine the prognosis of NSCLC.

Many studies have established their own diagnostic thresholds for the serum midkine levels to differentiate malignant and benign disease. Zhu et al. reported better performance for the serum midkine levels (cut-off value of 654 pg/ml) than the alpha-fetoprotein levels in detecting early-stage hepatocellular carcinomas [16]. Meng et al. proposed a cut-off value of 323.12 pg/ml to distinguish malignant from benign thyroid nodules, and the pre-131I-ablative serum midkine level was used to predict the presence of metastasis, with a sensitivity of 75.7% and a specificity of 75.0% [17]. Yamashita et al. determined an optimal cut-off value of 482 pg/ml using a ROC analysis to discriminate patients with head and neck squamous cell carcinoma from healthy individuals [13]. A slight increase in the serum midkine concentration may occur in benign pulmonary diseases because the midkine protein has multiple functions. However, there is a significant difference in the upregulation observed in the NSCLC group and the benign pulmonary disease group because rapid growth of malignant tumors often induces uncontrolled biological activities. Based on the reasons described above, our study has stressed the importance of setting the cut-off value. In previous reports, common methods used to achieve a cut-off value include Youden's index (sensitivity + specificity −1) and a distribution of values [13, 14]. The serum value of 320 pg/ml obtained with the maximum Youden's index yields 81.0% sensitivity and 74.7% specificity. Furthermore, the average serum midkine value of the healthy controls +2SD was 438 pg/ml, with 66.7% sensitivity and 93.2% specificity. Thus, in our study, we set a cut-off value of 400 pg/ml to predict NSCLC, with a sensitivity and specificity of 71.2% and 88.1%, respectively. While the specificity of malignancy was desirable, the sensitivity was somewhat low. The cut-off value is required to be 250 pg/ml to achieve 90.0% sensitivity, but the specificity was reduced to 32.2%. Therefore, a cut-off value of 400 pg/ml for midkine was used in the analysis to differentiate patients with NSCLC from patients with non-malignant pulmonary disease or healthy individuals. This value provided much better performance than other conventional tumor markers. This cut-off midkine concentration may be used as a promising pre-surgical biomarker to screen for NSCLC and may be appropriate for the clinical setting.

In our study, we observed significant correlations between the serum concentrations and tumor tissue expression of midkine. The IHC staining score of the tumor samples comprised the staining intensity and the percentage of positive IHC staining in the tumor tissues. Scores greater than 4 were defined as IHC-positive, and 400 pg/ml is considered the cut-off value for the serum midkine levels in patients with NSCLC. The mean IHC staining score in patients with high serum midkine values (serum midkine>400 pg/ml) was significantly higher than in the score in patients with low serum midkine values (serum midkine<400 pg/ml). A significant association was observed between the serum midkine levels and midkine immunoreactivity in tumor tissues. The serum midkine levels were significantly elevated in patients with an IHC staining score of 0 to 3 and an IHC proportion score of 0 to 4. The serum midkine levels were positively correlated with midkine expression in tumor tissues. Thus, the serum midkine levels were an appropriate substitute for midkine expression in tumor tissues from patients with NSCLC.

The serum midkine levels were elevated in patients with NSCLC, regardless of the clinicopathological features of the tumor, such as differentiation and TNM stage. Similar phenomena have been reported in other malignant tumors, including head and neck squamous cell carcinoma, esophageal squamous cell carcinoma, and hepatocellular carcinomas [13, 14, 16]. Thus, the increase in the serum midkine levels is observed at a relatively early stage in patients with NSCLC. Recently, Yuan et al. have proposed that overexpression of the midkine protein in NSCLC tumor tissues was positively correlated with the clinical stage [15]. In our study, although a significant correlation was observed between the serum midkine levels and midkine immunoreactivity in tumor tissues, no obvious association was observed between the serum midkine concentrations and the patients’ clinical features, possibly because the samples used for the IHC analysis were collected during surgical resection in patients without preoperative treatment. Patients in our cohort who received the surgical treatment must show no evidence of organ metastasis or distant lymph node metastasis, according to the NCCN guidelines. The patients’ clinical stages should be no more than stage IIIa, which is regarded an early stage of NSCLC. Midkine was reported to be a key molecule in carcinogenesis, angiogenesis, tumor growth and anti-apoptotic signaling [4]. Another possible reason why the distribution of midkine in peripheral blood might not always be consistent with its expression in tumor tissues is because the IHC results for midkine expression are presented as both staining intensity and the percentage of positive staining cells. However, the serum midkine levels determined using the ELISA are presented only as a concentration, which may lead to a loss of information. Although the reasons why the serum midkine concentrations do not parallel tumor progression remain to be elucidated, midkine may be very valuable as a convenient early detection biomarker in patients with NSCLC.

Urine was collected from 45 patients with NSCLC and 20 healthy controls at the same time as the blood samples. This study is the first to report a significant correlation between the serum and urine midkine levels in patients with NSCLC. The diagnostic value of the serum midkine concentrations in detecting several malignant tumors during their early stages has been discussed [13, 16]. As a secreted protein, midkine can be detected in body fluids such as cerebrospinal fluid [18]. Moreover, urinary midkine concentrations are elevated in various human tumors, such as gastric, colon and hepatocellular carcinomas [10]. In agreement with previous findings, the urine midkine levels were significantly increased in patients with NSCLC, with an average value of 1000 pg/mg Cr. Additionally, the average urine midkine level was 226 pg/mg Cr in healthy controls, which was similar to the value reported in a previous study [10]. If a correlation between the serum and urine midkine levels can be established, the urine midkine level will be a method to detect individuals at high risk of developing some malignant tumors using a routine health examination. Compared with other biomarkers, urinary biomarkers are convenient and non-invasive tools for the systematic and large-scale screening of tumors.

In our study, 110 patients with NSCLC who had received radical lobectomy and systematic lymphadenectomy were followed for 5 years. The serum midkine levels were significantly associated with the overall survival of patients with NSCLC and with the T classification, N classification and clinical stage. The survival rate in the high-serum midkine group was remarkably lower than the low-serum midkine group in our cohort. Furthermore, the serum midkine levels were an independent prognostic factor in the multivariate analysis of patients with NSCLC. Although the serum midkine level does not depend on tumor progression, the suppression of midkine expression inhibited lung cancer cell growth *in vitro* and *in vivo* by inducing apoptosis and inhibiting the PI3K signaling pathway [19]. Thus, midkine may be a good indicator for identifying high-risk patients and a therapeutic target for lung cancer.

Further research is required due to two limitations in this study. The unexpected finding of the lack of correlation between the serum midkine levels and tumor progression in patients with NSCLC may be because of the relatively small number of patients with NSCLC who were recruited in our cohort. As a candidate cancer biomarker, the surveillance function of the serum midkine levels is very important because the levels are predicted to decrease after curative surgery or increase after relapse [16]. In our study, we investigated the serum midkine concentrations of patients before they received any type of treatment and followed the 5-year survival of only the patients who received surgery. Based on a new report, the serum midkine levels may reflect chemosensitivity in patients with head and neck squamous cancer [13]. Additional studies of the serum midkine levels in patients who received surgery or chemotherapy are required. Furthermore, a large cohort of patients is required to investigate the value of the urine midkine levels as a new approach for the large-scale screening of high-risk individuals. We are currently pursuing these two lines of investigation.

In summary, the serum midkine levels in patients with NSCLC were an independent prognostic factor, consistent with the expression of the midkine protein in NSCLC tissues. Therefore, serum midkine levels or even urine midkine levels may be useful, minimally invasive biomarkers for detecting and predicting the prognosis of NSCLC.

## MATERIALS AND METHODS

### Patients and controls

In this study, 153 patients with NSCLC who had not been treated with any type of therapy were recruited from the Department of Thoracic Surgery at The First Affiliated Hospital of Sun Yat-Sen University from September 2010 to September 2011. The patients consisted of 101 males (66%) and 52 females (34%) with a median age of 59 years (range: 39-73 years) and were classified according to the 7th edition of the AJCC Cancer Staging Manual. Two pathologists independently provided the histopathological diagnosis for all samples. According to the National Comprehensive Cancer Network (NCCN) guidelines, 110 patients who had undergone radical lobectomy with systematic lymphadenectomy and 43 patients who had distant lymph node or organ metastasis received chemotherapy. Patients who received surgery had stage I, II and well-selected IIIa tumors, such as T3N1M0, according to the TNM classification. Depending on the results obtained from the frozen section during the operation, the operation record and post-operative microscopic examination of the resection margins, surgeries were conducted as macroscopically complete removal of the tumor, known as R0 resection. The median follow-up period for all surgery patients was 60 months (range: 6 to 60 months).

Ninety-five healthy individuals and 23 patients with benign pulmonary disease were also investigated as controls. Patient recruitment and sample collection were conducted within the guidelines of protocols approved by the institutional review boards. Informed consent was obtained from all of the subjects. Individuals who had malignant disease within 5 years were excluded as the control cases. Healthy individuals consisted of 58 males (61%) and 37 females (39%) with a median age of 54 years (range: 21-75 years) and without evidence of comorbid diseases (e.g., esophagitis, liver dysfunction, diabetes, etc.), as previously described. The patients with benign pulmonary disease consisted of 14 males and 9 females with a median age of 59 years (range: 28-78 years), who served as non-cancer controls. This group included individuals with pulmonary hamartoma (8 cases), pulmonary tuberculoma (7 cases), intrapulmonary hematoma (4 cases), pulmonary cryptococcosis (3 cases), and papilloma (1 case).

This study was performed in accordance with the Declaration of Helsinki and was approved by the Ethics Committee of The First Affiliated Hospital of Sun Yat-Sen University. All of the patients and controls provided written informed consent to participate in this study.

### Serum midkine and urine midkine measurements

After obtaining written informed consent, blood and urine were collected from patients and controls. Following a 12-hour overnight fast, venous blood samples were collected from all subjects and centrifuged at 5,000 *g* for 5 min. The urinalysis was performed by collecting morning midstream samples from 45 patients with NSCLC and 20 healthy controls. A volume of 5.0 ml of urine was collected from each participant. Serum and urine were collected, stored frozen at -80°C, and then thawed on ice prior to the analyses. An ELISA kit for human midkine (BioRay Inc., Norcross, WA, USA) was used to detect the serum and urine midkine concentrations. We determined the serum and urine midkine levels in the collected samples according to the manufacturer's protocol. Briefly, 100-μl urine samples were added to each well and incubated for 2.5 hours at room temperature. After incubation, the plates were washed 4 times with wash buffer, and then 100 μl of the prepared biotinylated antibody was added to each well and incubated for 1 hour at room temperature. After washing 4 times, 100 μl of the prepared Streptavidin solution was added to each well and incubated for 45 minutes at room temperature. After washing 4 times, 100 μl of TMB One-Step Substrate Reagent was added to each well and then the plate was incubated in the dark for 30 minutes at room temperature. The reaction was finally stopped by the addition of 50 μl of Stop Solution. The optical density (OD) was measured at 450 nm in a micro-plate reader (Spectramax m5/m5e; Molecular Devices, LLC, Sunnyvale, CA, USA). A creatinine kit (Beckman Coulter Inc., Miami, FL, USA) was used to measure the creatinine concentrations in urine according to the manufacturer's instructions.

### Immunohistochemistry

IHC was performed using the surgically excised specimens from 72 patients with NSCLC to compare the expression of the midkine protein in the tumor tissues with the serum midkine concentrations. The specimens were fixed with 15% formaldehyde for more than 48 h, and paraffin-embedded tissue blocks were prepared. IHC was performed on 4-*μ*m-thick tumor sections from these paraffin blocks. Heat-induced antigen retrieval was performed on deparaffinized sections in 10 mmol/l citrate buffer (pH 7.25) for 10 min at 100°C. Endogenous peroxidase activity was blocked by incubating the sections in 3% hydrogen peroxide in methanol for 15 min. The primary antibody was an anti-midkine rabbit monoclonal antibody (1:200 dilution; Abcam, Cambridge, MA, USA). The slides were incubated with the primary antibody overnight at 4°C. After washing with phosphate-buffered saline, the tissue sections were incubated with a biotinylated secondary antibody and streptavidin-horseradish peroxidase complex, each for 30 min at room temperature. Subsequently, the nuclei were counterstained with hematoxylin (Abcam; Cambridge, MA, USA). Negative control sections were incubated with phosphate-buffered saline (PBS) instead of the primary antibodies.

Midkine immunoreactivity was quantitatively scored according to the percentage of positive cells and staining intensity. The staining intensity was graded as follows: negative=0; weak=1; moderate=2; and strong=3. The percentage of positively stained cells was scored as follows: <5%=0; 6-25%=1; 26-50%=2; 51-75%=3; and >76%=4. The final score was determined by multiplying the staining score and proportion score (intensity score × proportion score). The final staining score ranged from 0 to 12. For the statistical analysis, final staining scores of 0-4 and 6-12 were designated as low and high expression, respectively.

### CEA, NSE and CFRA21-1 assays

CEA (reference 0.00-0.50 μg/l), NSE (reference 0.00-16.30 ng/ml) and CYFRA21-1 (reference 0.00-3.30 ng/ml) assays were performed on a fully automated ADVIA Centaur analyzer (Siemens Healthcare Diagnostics, New York, USA). These assays were based on the chemiluminescent reaction principle.

### Statistical analysis

SPSS 20.0 for Windows software system (SPSS Inc., Chicago, IL) and GraphPad Prism 5 (Version X; La Jolla, CA, USA) were used for the statistical analyses. Differences between the groups were analyzed using the Mann-Whitney *U* test and Fisher's exact test. Univariate ANOVA analysis and the least significant differences (LSD) test was used to compare the means of more than two independent groups. The cut-off values for the serum midkine levels to predict the presence of NSCLC were determined using ROC curves. The overall survival rates of patients with NSCLC were calculated using the Kaplan–Meier method and were analyzed using the log-rank test. The multivariate analysis was performed using Cox's proportional hazards regression model. Differences or correlations with *P* values <0.05 were considered statistically significant.

## SUPPLEMENTARY FIGURES


