# Supplementary material for: Midkine is a serum and urinary biomarker for the detection and prognosis of non-small cell lung cancer
Source: Oncotarget. 2016 Dec 10;7(52):87462–72. doi: 10.18632/oncotarget.13865 (PMC5350001; doi:10.18632/oncotarget.13865)
Supplement: Supplementary file 1 [file oncotarget-07-87462-s001.pdf]

Midkine is a serum and urinary biomarker for the detection and prognosis of non-small cell lung cancer

SUPPLYMENTARY FIGURES

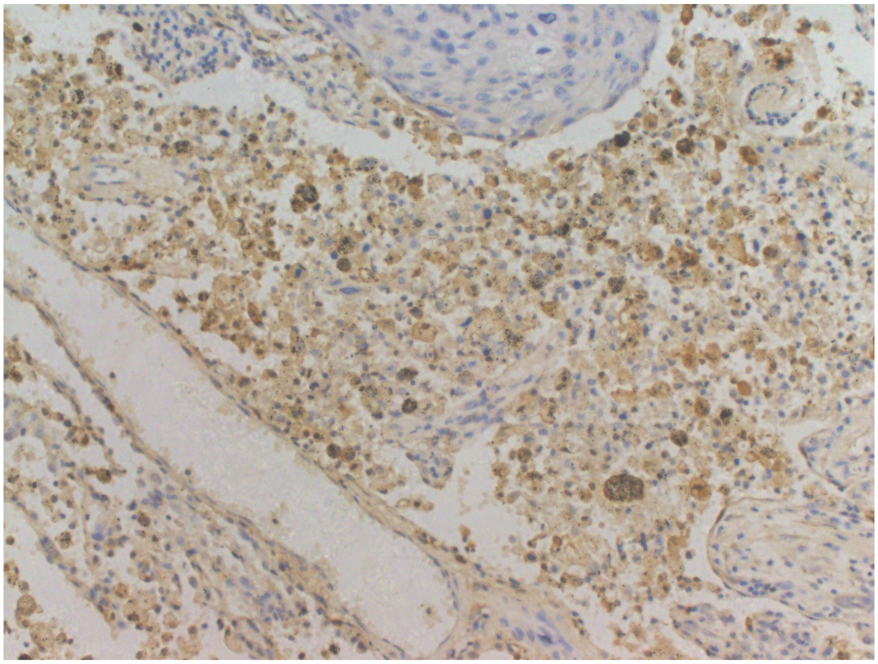

Supplementary Figure S1: Representative IHC result of tumor specimen. The intensity score was 3. The proportion score was 4. The mean IHC score was 12.

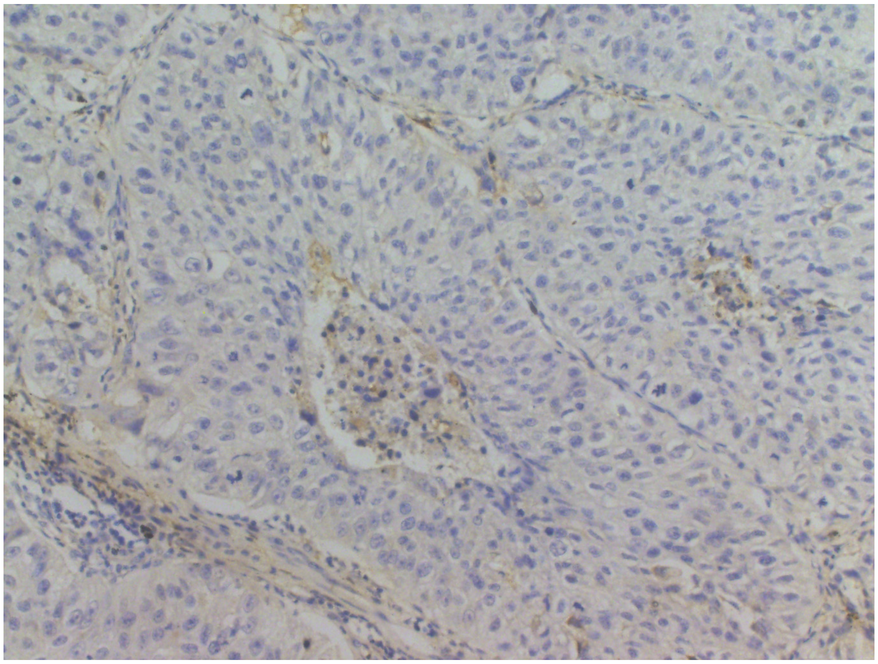

Supplementary Figure S2: Representative IHC result of tumor specimen. The intensity score was 1. The proportion score was 1. The mean IHC score was 1.

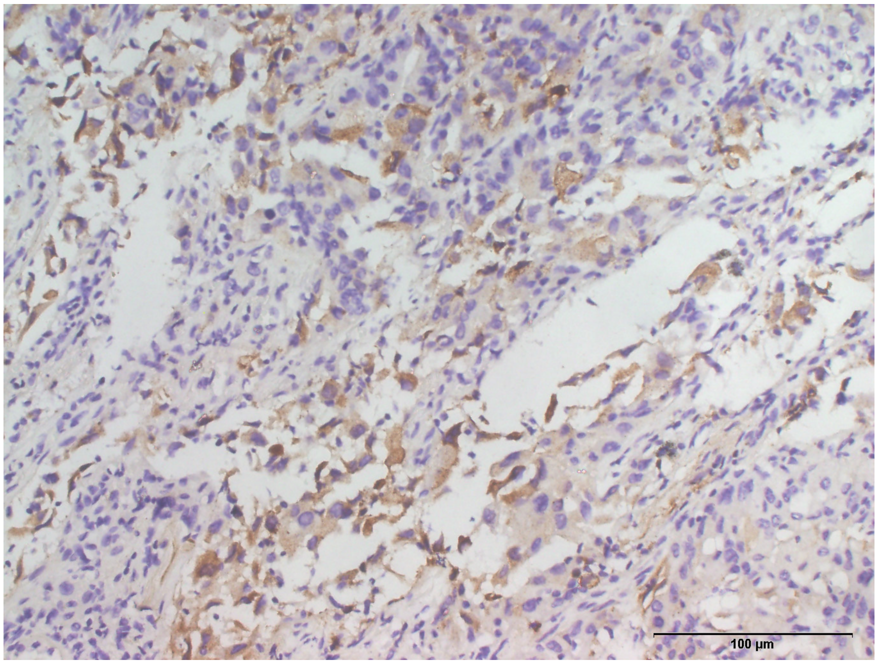

Supplementary Figure S3: Representative IHC result of tumor specimen. The intensity score was 3. The proportion score was 1. The mean IHC score was 3.

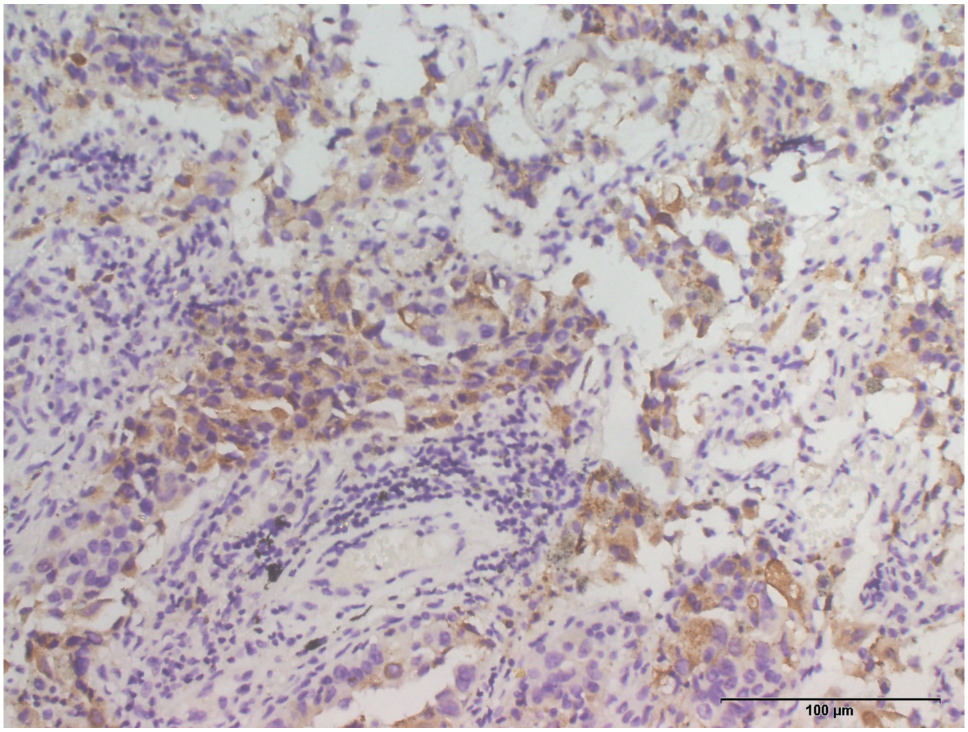

Supplementary Figure S4: Representative IHC result of tumor specimen. The intensity score was 3. The proportion score was 2. The mean IHC score was 6.

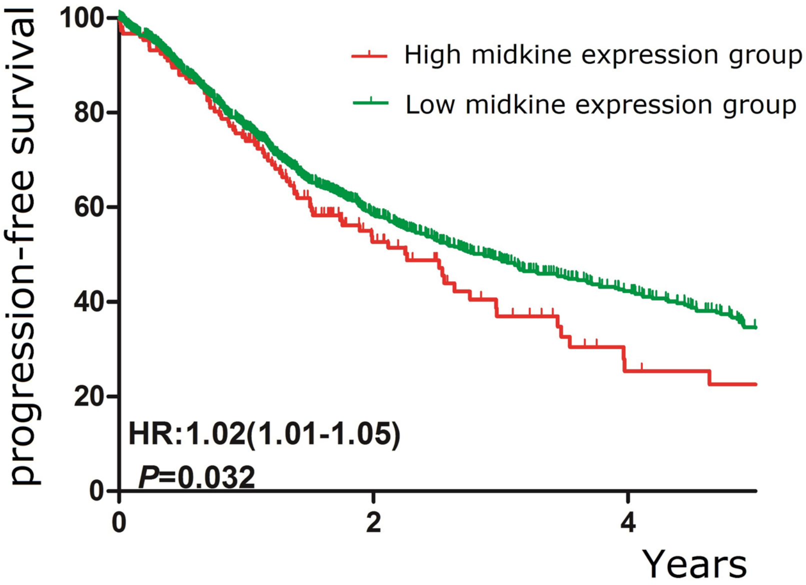

Supplementary Figure S5: Relationship between midkine expression in tumor tissues and patient progression free survival (PFS) rate using The Cancer Genome Atlas (TCGA) lung squamous cell carcinoma and lung adenocarcinoma cohort.
